# Natural enemies partially compensate for warming induced excess herbivory in an organic growth system

**DOI:** 10.1038/s41598-017-07509-w

**Published:** 2017-08-04

**Authors:** Orsolya Beleznai, Jamin Dreyer, Zoltán Tóth, Ferenc Samu

**Affiliations:** 1 Department of Zoology, Plant Protection Institute, Centre for Agricultural Research, Hungarian Academy of Sciences, Budapest, Nagykovácsi út 26-30 H-1029 Hungary; 2 Department of Entomology, University of Kentucky, S-225 Ag Science Center N Lexington, Kentucky, 40506-0091 USA; 3 Lendület Evolutionary Ecology Research Group, Plant Protection Institute, Centre for Agricultural Research, Hungarian Academy of Sciences, Budapest, Herman Ottó út 15 H-1022 Hungary

## Abstract

Predators can limit prey abundance and/or levels of activity. The magnitudes of these effects are contingent on predator and prey traits that may change with environmental conditions. Aberrant thermal regimes could disrupt pest suppression through asymmetric effects, e.g. heat-sensitive predator vs. heat-tolerant prey. To explore potential effects of warming on suppressing pests and controlling herbivory in a vegetable crop, we performed laboratory experiments exposing an important pest species to two spider predator species at different temperatures. Heat tolerance was characterised by the critical thermal maxima parameter (CTM50) of the cucumber beetle (*Diabrotica undecimpunctata*), wolf spider (*Tigrosa helluo*), and nursery web spider (*Pisaurina mira*). Cucumber beetles and wolf spiders were equally heat tolerant (CTM50 > 40 °C), but nursery web spiders had limited heat tolerance (CTM50 = 34 °C). Inside mesocosms, beetle feeding increased with temperature, wolf spiders were always effective predators, nursery web spiders were less lethal at high temperature (38 °C). Neither spider species reduced herbivory at ambient temperature (22 °C), however, at warm temperature both species reduced herbivory with evidence of a dominant non-consumptive effect. Our experiments highlight the contingent nature of predator-prey interactions and suggest that non-consumptive effects should not be ignored when assessing the impact of temperature change.

## Introduction

Predators can negatively impact prey populations either by directly consuming prey individuals or through non-consumptive effects (NCEs) that trigger costly anti-predator reaction in prey^1^. The wide ranging behavioural responses of prey to the presence or cues of predators may result in decreased feeding^2^, lower quality diet^3, 4^ or physiological stress^5^. Predator effects have the potential to cascade through ecological systems, affecting productivity and ecosystem functioning at the lower levels of the food web^6^ and shaping ecological interactions spatially^7^. Elements at both sides of this interaction, predator activity, performance and the success or failure of the prey’s anti-predator behaviour are contingent on environmental factors. These effects gain economic importance in the natural enemy – pest context, especially in the view of global climate change.

With climate change both mean summer temperatures and temperature variability are projected to increase^8^. This results in the more frequent occurrence of climate extremes, such as heatwaves^9^, which may have particularly large impact on ecological systems^10^. Studies in natural ecosystems^11, 12^ suggest that the outcome of predator-prey interactions can be especially temperature sensitive, because differences in temperature tolerance of the interacting species can magnify the response^13^. In agroecosystems natural enemy – pest interactions are in the focus of biological control research since any shift in the efficiency of biological control processes has paramount economic importance. Not only expected elevated temperatures arising from global climate change, but also high temperatures caused by some agricultural practices, such as greenhouse growing^14^, plastic mulch and foil covers^15, 16^ make it necessary to study how different interactions may change under altered thermal conditions. Research has been done on how plant-herbivore interactions are affected by rising temperatures, reviewed by Bale *et al*.^17^. More recently, studies have addressed the question of how herbivore – natural enemy interactions might respond to climate change^18, 19^, but we still need more studies to understand complex, multitrophic and non-consumptive effects of rising temperatures^20, 21^. The present study examines how a ‘crop - herbivore pest – generalist predators’ system responds to temperature changes.

The tritrophic system of ‘organically grown cucumber crop –cucumber beetle – two species of spider natural enemies’ is a highly relevant model system for studying the temperature sensitivity of trophic interactions. Despite the importance of cucurbit crops, organic farmers largely avoid growing them because feeding damage by insect pests and spread of pathogens can account for as much as 80% loss in the marketable product^22^. One of the major pests is the spotted cucumber beetle, *Diabrotica undecimpunctata howardi* (Barber, 1947). Both organic and conventional growers rank the cucumber beetle/bacterial wilt complex as one of the most important and least controllable threat to cucumber crop^23^. Current organic management options include using polypropylene fabric row covers to keep out pests, and organic insecticides combined with proper planting dates^24^. Row covers applied in this practice highly increase crop temperature.

Natural enemies, including spiders, have been shown to be important in organic cucurbit systems because early in the season they were able to reduce the densities of striped cucumber beetles and cause fruit production to increase^25, 26^. As a result of the NCE of wolf spiders, cucumber beetles were shown to modify their behaviour, including a reduced feeding activity on plants, which resulted in nearly 50% reduction in feeding damage^27, 28^. Since spiders are effective predators and biological control agents, some aspects of the temperature dependence of their direct effect and NCE on prey have already received some attention in the literature. In Barton’s^29^ study of grassland ecosystems, warming had no direct effect on herbivores, but it did decrease spider activity which caused a reduced predation risk for herbivores. Lubin and Henschel^30^ also demonstrated that in spiders high daytime temperatures may restrict the time available for foraging. At the same time, higher temperatures can increase herbivore activity, translating to a greater amount of host plant consumption^17^. The interaction of spider and herbivore thermal responses is exemplified in an old field case study where grasshoppers could reduce predator encounters by becoming more active during the hottest parts of the day when spiders reduced their activity to avoid thermal stress^31^. The outcome of such interactions depends on the individual thermal tolerances of the constituent species. Since different species within the same trophic level can have different tolerances, complementarity may play an important role in food webs with more species rich predator component^32, 33^. In accordance with that, in a recent experimental study of the natural enemy complex of grapevine pests synergistic effects between predators have been shown to increase under increased temperatures^34^.

Our main question was whether warming will increase or dampen predator effect in a tritrophic system. In the studied organically grown cucumber system elevated temperatures pose an acute problem both as a result of summer heat waves, projected to be more frequent due to global warming, and because of current management practices relying on different heat accumulating covers. We analysed how two spider species, with differential responses to warming, interacted with spotted cucumber beetles at different temperatures, and how these interactions finally affected cucumber plant damage. Our initial prediction was that spiders would increase beetle mortality, change beetle activity and decrease damage to plants. We predicted that temperature will raise the activity of the prey, but will also increase predator effect with a net effect of decreased plant damage. We tested our predictions by (i) establishing thermal tolerance of spiders and cucumber beetles over a wider temperature gradient; (ii) by analysing the temperature dependence of the tritrophic interaction, i.e. predator effect on beetle mortality, activity and plant damage.

## Methods

### Collecting and keeping study subjects

The experiments were conducted on individually potted squash plants (*Cucurbita maxima* (Duchesne, 1786), Thiram-treated “Blue Hubbard” variety). Squash plants were grown in 11 cm diameter pots in the Department of Entomology greenhouse at the University of Kentucky (1100 South Limestone St, Lexington, KY 40546) under a 16:8(L:D) h photoperiod at 25 °C. Plants selected for experimental trials were similar in size and identical in age, with 2 cotyledons and 2 true leaves fully unfurled.

The spotted cucumber beetle, *Diabrotica undecimpunctata howardi* (Barber, 1947) is a polyphagous herbivore^35^. Its larvae feed on roots and the adults feed on plant foliage and flowers^36^. Cucumber beetles were field collected from research and commercial fields surrounding Lexington, Kentucky USA during September 2014. Beetles were captured from pumpkin and squash foliage by sweep nets, aspirated, and placed inside clear plastic containers (11 cm diam., 15 cm high). The mesh-topped containers were provisioned with fresh water and cucurbit leaves and kept at room temperature (22 °C) and a 18:6 (L:D) h schedule in the laboratory.

In the experiments we used two key natural enemy species of the cucumber beetle. The wolf spider *Tigrosa helluo* (Walckenaer 1837) (Araneae, Lycosidae) is relatively large predatory arthropod (adult body length 15–20 mm), a dominant spider in agro-ecosystems which is active throughout the summer^37^. The nursery web spider *Pisaurina mira* (Walckenaer 1837) (Araneae, Pisauridae), is a sit-and-wait ambush predator, one of the most common spiders in the eastern United States, and can be an important natural enemy in crops^38^. Spiders were hand collected at night from the University of Kentucky North Farm research facility (1925 Research Farm Road, Lexington, KY USA) during September 2014. Spiders were held at room temperatures and at 18:6 (L:D) h schedule in individual plastic containers (7 cm diam., 5 cm high) with wetted cotton balls. They were fed *Drosophila melanogaster* (Meigen, 1830) prey *ad libitum* twice a week. Prior to use in an experiment, individually numbered spiders were starved for 2 days, weighed, and inspected. Animals were used in the experiments within 1 month of their collection.

### Critical Thermal Maxima of the species

To characterize the heat tolerance of the beetle and the spider species, we determined their critical thermal maxima. The original concept of the critical thermal maximum was introduced by Cowles and Bogert^39^ and was defined as “the thermal point at which locomotory activity becomes disorganized and the animal loses its ability to escape from conditions that will promptly lead to its death.” From 20 individuals of each species, kept individually in small clear plastic containers, we randomly assigned ten to a control group constantly held at room temperature, while the other ten received a treatment of temperature increase. Inside a climate-controlled growing chamber, the temperature of these individuals was increased by approximately 1 °C every 5 minutes, beginning at 21 °C and continuing until 48 °C. Temperature inside the chamber was measured using a HOBO (HOBO U23 Pro v2 Temperature/Relative Humidity Data Logger - U23-001-ONSET) data logger at one-minute intervals. We recorded the response of each individual at five-minute intervals, scoring any purposeful body movement (e.g. attack, running, walking) as a response. To differentiate inactivity from non-responsiveness, wolf spiders were prodded with a wooden probe while containers holding escape-prone beetles and nursery web spiders were gently agitated. Oftentimes, as individuals became non-responsive their bodies showed signs of extreme heat stress and/or death (e.g. contraction of legs). The experiments were performed on 13 October 2014. Collected data was used to statistically characterise the critical thermal maxima of the species by calculating the CTM_50_ parameter, which indicates the temperature at which 50% of the individuals was unresponsive.

### Temperature effect on squash – cucumber beetle – spider tritrophic system

We conducted a fully-crossed 2 × 3 experiment with two levels of temperature and three levels of predator presence (‘predator treatment’) to determine the interactive effect of temperature and predators on cucumber beetle mortality, activity and on plant damage. We ran trials in five replications for the spider treatments × temperature levels combinations (n = 20) and in 10 replications for the control × temperature levels combination (n = 20). Trials were conducted in mesocosms with a single squash plant. To create the mesocosms, acrylic glass cylinders (10.5 cm diameter, 30 cm tall) with fine mesh windows and top were placed over the top of the squash plants in the pot, with the lower edge of the cylinder buried 1–2 cm deep into the soil. We had n = 20 trials at 22 °C and n = 20 trials at 38 °C. Trial mesocosms were kept in climate-controlled chambers of the above temperatures, which were monitored at one-minute intervals inside the chambers using a HOBO data logger.

For each trial three cucumber beetles were placed inside a mesocosm. At both temperatures we had three levels of predator presence: (i) control with no predator, (ii) a wolf spider ‘lycosid’ present or (iii) a nursery web spider ‘pisaurid’ present. In trials with spider predators, after a 15 minute beetle acclimation period (beetles stayed in the mesocosms without predators) cylinders were tilted briefly to allow the rapid introduction of a single spider at ground level (Supplementary Fig. 1). Mesocosms were checked at one-hour intervals between t = 0 and t = 6 hours, and the survival, position and feeding activity of the three cucumber beetles was recorded. Levels of position could be on the soil surface or on the plant stem, below the leaves, constituting the category “low”, or on or above the lower leaf, constituting the category “high”. Levels of feeding were “feeding” (actively consuming plant material) or “not feeding”. The plant damage on both leaves and the stem was quantified by the assessed percentage of leaf area and percentage of the stem volume affected by beetle bites (0% = no damage, 100% = complete damage). Mesocosm experiments were performed on 14–16 October 2014.

### Statistical analysis

All analyses were conducted in R v. 3.3.2^40^. We compared the thermal response curves of the three arthropod species in the warming chambers using a generalized linear mixed model with a binomial distribution and using a logit link function (‘glmer’ function in the ‘lme4’ package^41^), with arthropod species as a factor and individual specimen as a random effect. From the estimated coefficients of the modelled logistic curves we calculated CTM_50_ values and their confidence intervals^42^, which gave the temperature at which 50% of the individuals are expected to be unresponsive.

In the tritrophic system we considered the following responses: beetle mortality, beetle activity and plant damage. Mortality was represented by the ratio of dead animals by the end of the trial. We had three response variables for beetle activity: (i) the ‘feeding ratio’ (FR) represented the ratio of alive beetles actually feeding at each hourly observation; (ii) the ‘mean feeding ratio’ (MFR) was mean of FR values averaged across the hourly observations; (iii) the ‘mean position ratio’ (MPR), the ratio of alive beetles of position high in the mesocosms at each hourly observation, averaged across observations (MPR = 1 if all beetles are of position high, MPR = 0 if all beetles are of position low). The response variable ‘plant damage’ was derived as the mean percentage of damage on the two leaves and on the stem.

Both plant damage and MFR were modelled in a linear model with temperature and predator treatment and their interaction as explanatory factor variables. In the case of MFR the response variable was square root transformed. In the case of beetle mortality and MPR we had zero variance for certain treatment combinations (all animals survived in 22 °C control and all beetles were of position low at 38 °C with lycosid treatment); in these cases we split the data and analysed predator treatment effects separately for 22 °C and 38 °C. For mortality at 22 °C and MPR at 38 °C we applied Kruskal-Wallis test, with Dunn post-hoc test with Holm probability adjustment. Mortality at 38 °C was modelled by generalized linear model with binomial distribution and using a logit link function. Furthermore, we tested for difference in spontaneous mortality between temperatures within the control group by Fischer’s exact test. MPR at 22 °C was modelled with a linear model including predator treatment as the only explanatory variable. Temperature dependent temporal trend in the feeding activity (repeated measures FR data) was modelled using generalized estimating equations (GEE) with binomial distribution (‘geeglm’ function of the ‘geepack’ package^43^). Into this model, we included temperature, predator treatment, hour and the interaction between hour and temperature, and also controlled for temporal autocorrelation^43^. Model fit in all cases was checked graphically, by inspecting residuals and their QQ plots. Significance of modelled effects was tested with either the built-in ‘anova’ or with the ‘Anova’ function of the ‘car’ R package^44^ with type 3 analysis of variance. Significance of the difference between treatment levels was assessed using multiple comparisons of means with Tukey contrasts using the ‘lsmeans’ R package^45^.

### Ethics statement

All applicable international, national, and/or institutional guidelines for the care and use of animals were followed. All procedures performed in studies involving animals were in accordance with the ethical standards or practice of the institution at which the studies were conducted.

## Results

### Critical Thermal Maximum

**Figure 1:**
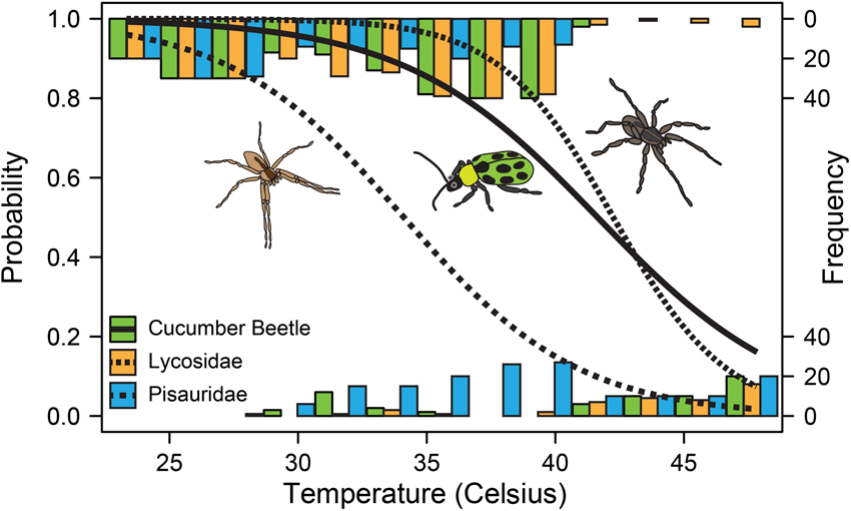
Fitted logistic functions of arthropod thermal tolerance for three species. The spotted cucumber beetle *D. undecimpunctata* (solid line), wolf spider *H. helluo* (darker spider, small dashed), and nursery web spider *P. mira* (light brown spider, large dashed). Bars give the frequency distribution of responsive (top) and unresponsive animals (bottom) binned by 2 °C increments (bar colours: cucumber beetle green, pisaurid blue, lycosid orange).

Arthropods in the constant ambient temperature control (temperature: mean = 21.4 **°**C, S.D. = 0.11 **°**C) showed no difference in response over time, all three species responded actively at every single inspection, therefore a statistical model was not fitted. However, animals became unresponsive at various temperatures exceeding 30 °C in the warming chambers. The fitted generalized linear mixed model indicated that thermal responses were highly significantly different between the study animals (type 3 Wald χ^2^ test, effect of species: χ^2^ = 92.75, d.f. = 2, P < 0.0001). The calculated critical thermal maximum parameters indicated that cucumber beetles and the lycosid spider had high heat tolerance, with largely overlapping confidence intervals (cucumber beetle: CTM_50_ = 41.8 **°**C, 95% CI [40.48, 43.16]; lycosid: CTM_50_ = 44.3 **°**C, 95% CI [42.70, 45.85]), while the pisaurid spider proved to be much less heat tolerant (CTM_50_ = 34.2 **°**C, 95% CI [33.19, 35.15]) (Fig. 1).

### Tritrophic system

#### Mortality

**Figure 2:**
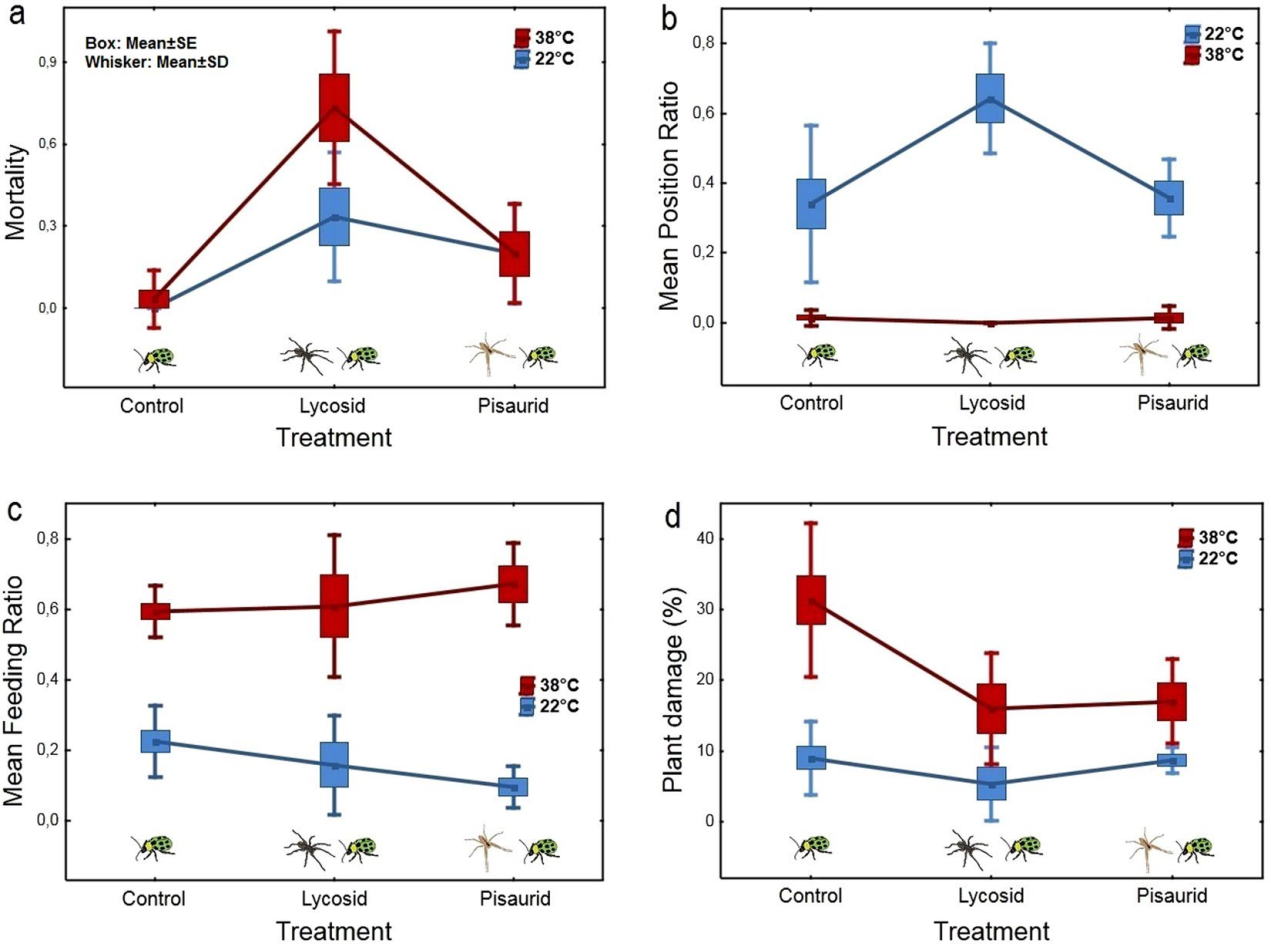
Cucumber beetle responses. Mean cucumber beetle responses in mortality (**a**), Mean Position Ratio (MPR) (**b**), Mean Feeding Ratio (MFR) (**c**) and Plant Damage (**d**) to the three predator treatments (control, lycosid, pisaurid) under the two different temperatures (22 °C, 38 °C). Response variables Mortality, MPR and MFR represent the mean ratio beetles in a trial falling prey, occupying position ‘high’ and showing feeding activity, respectively. Plant damage is the mean percentage of damage on the two leaves and the stem of cucumber plants by the end of the experiment. For further explanation see text.

No difference was found in the spontaneous beetle mortality (only control cases considered) between the two temperatures. We had zero mortality at 22 °C in 10 trials (n = 30 beetles) and only one beetle died during the experiment at 38 °C in the same number of trials (Fischer’s exact test, P = 1.0). We could consider the effect of spiders on beetle mortality only separately in the two temperature groups (due to zero variance in some treatment combinations). At 22 **°**C overall predator treatment effect was significant (Kruskal-Wallis test: χ^2^ = 10.69, d.f. = 2, P = 0.005; Fig. 2a), and according to Dunn’s post-hoc test both the lycosid treatment (P = 0.003) and the pisaurid treatment (P = 0.03) caused significantly higher beetle mortality than in the control. At 38 °C a similar pattern emerged; the overall predator effect was highly significant (ANOVA on the binomial generalized linear model, effect of predator treatment: LR χ^2^ = 26.30, d.f. = 2, P < 0.0001; Fig. 2a), but that was only attributable to lycosids, which caused the highest mortality, significantly different from control and pisaurid effect (contrasts: lycosid-control: z = 3.73, P = 0.0005; pisaurid-lycosid: z = −2.75, P = 0.01).

#### Activity

Beetles had a differential preference for strata between the temperature treatments (effect of temperature on MPR: χ^2^ = 29.71, d.f. = 1, P < 0.0001), taking higher positions at 22 °C and lower positions at 38 °C (Fig. 2b). The effect of predator treatment was also significant for MPR (χ^2^ = 9.39, d.f. = 2, P = 0.009), which according to the post-hoc test could be ascribed to the effect of lycosid spider at 22 °C (contrasts: control – lycosid: t = −4.13, d.f. = 34, P = 0.0006; lycosid – pisaurid t = 3.37, d.f. = 34, P = 0.005), meaning that beetles were higher on the cucumber plants when a lycosid spider was present. However, at the higher temperature there was no significant predator effect (Fig. 2b).

Feeding activity was highly significantly influenced by temperature (F = 51.7, d.f. = 1, 34, P < 0.0001), as beetles showed feeding activity much more frequently at the higher temperature treatment (Fig. 2c). Predator presence also had significant, albeit weaker effect on MFR (F = 4.29, d.f. = 2, 34, P = 0.02), which, according to the post-hoc test, manifested in the single significant effect of the pisaurid spider at 22 °C, which decreased feeding activity compared to control (t = 2.78, d.f. = 34, P = 0.02). At 38 °C feeding activity was not influenced by predator presence. Overall predators had marginally significantly different effect at the two temperatures (effect of temperature × predator interaction: F = 2.72, d.f. = 2, 34, P = 0.07). Hourly observations of FR indicated that there was a slight tendency of declining feeding activity over the 6 hours observation (effect of hour in geeglm model: χ^2^ = 3.4, d.f. = 1, P = 0.07); however, this trend was not different at the two temperatures (effect of temperature × hour: χ^2^ = 0.1, d.f. = 1, P = 0.75).

#### Plant damage

Plant damage was significantly higher at 38 °C than at 22 °C (Fig. 2d, effect of temperature: F = 46.75, d.f. = 1, 34, P < 0.0001). Predator treatment had no overall significant effect (F = 0.44, d.f. = 2, 34, P = 0.64), but the significant predator × temperature interaction (F = 3.92, d.f. = 2, 34, P = 0.03) indicated that spider presence had differential effect at different temperatures. At 38 °C the presence of either spider species caused a significant reduction in plant damage (control – lycosid: t = 3.83, d.f. = 34, P = 0.001; control – pisaurid: t = 3.58, d.f. = 34, P = 0.003), while at 22 °C none of the treatment comparisons were significant. The effect of the two spider species did not differ significantly from each other at either of the temperatures (Fig. 2d), and we cannot say that adding spiders to the system would diminish temperature effect on plant damage, since considering only non-control cases temperature effect was still significant (F = 14.84, d.f. = 1, 17, P = 0.001), although to a lesser degree than in the case of control cases alone (effect of temperature: F = 34.38, d.f. = 1, 18, P < 0.0001).

## Discussion

Both warming and predators affected the studied tritrophic system, in many aspects involving interactions between temperature and predator effect and ultimately influencing herbivore damage to plants. These interactions stem from the differential responses of the cucumber beetles and the two spider species to high temperatures. The examined thermal tolerance (CTM_50_) values can be regarded as proxies for threshold temperatures, where predator and prey activity critically change in a way that it affects the outcome of species interactions^13^. We had the expectation that in the studied cucurbit system niche partitioning along vegetation strata will determine the thermal tolerance of the interacting species, because vegetation top receives more irradiation, while lower strata are more shaded and also more humid. Accordingly, we expected that cucumber beetle and the pisaurid spider which are active in the crop canopy^23, 38^ should have higher tolerances and keep up activity under higher temperatures. Conversely, for sit-and-wait predators with permanent burrows, such as the studied lycosid spider *T. helluo*, the risks of exposure to temperature extremes are reduced^46^, and these animals would be expected to have a lower heat tolerance than a non-burrowing predator in the same environment^30^. However, contrary to our expectations, we received a different order of heat tolerance. The cucumber beetle and the lycosid spider had very similar and rather high tolerances to elevated temperatures, while the canopy living pisaurid *P. mira* was the most vulnerable to heat stress and the less effective predator at warmed temperature. Even though not meeting expectations about thermal sensitivity order, this scenario also presented the opportunity to study interactions between prey and predator of similar and different heat tolerances.

While both predators increased beetle mortality at low temperature, at high temperature only the heat tolerant lycosid predator could increase mortality, the heat sensitive pisaurid caused negligible mortality on prey. Within the operational thermal range metabolic rates, and consequently biological activity increases with temperature^47^, prey and predator activity are both expected to increase. For example, Dell *et al*.^48^ reported that organisms have a steeper thermal response for voluntary movements, such as foraging, than when trying to avoid predators. Therefore, it can be hypothesized that the performance curves of prey and predators often intersect, and as a consequence the predators’ attack success may vary from being high at temperatures where they can outperform their prey, to low at temperatures where they perform worse. This means that, as temperature changes, the ability of a predator to catch prey will decrease, increase, or remain unchanged depending on the relative effect of temperature on predator and prey^49^. Differences in thermal acclimation^50^ to temperature changes could also affect the cascading effect of predators in a multitrophic system^51^, even though in the present system activity changes were found to be similar at the two temperature regimes, suggesting a minor importance of the acclimation effect. We suggest that the two spider predators had such contrasting mortality effects, because for the lycosid the elevated temperature regime meant performance increase, while for the pisaurid it meant a decrease in predator effectivity.

As most environments show diurnal and seasonal thermal variations, multiple predators with different thermal responses are able to exert predation pressure over wider temperature ranges. Multiple predator effects^52^ or MPEs can modify the strength of pest regulation, causing positive or negative deviations from those that are predicted from independent effects of isolated predators^53, 54^. MPEs can be especially delicate, when – like in our case - there is a possibility of intraguild predation or intraguild NCE^53^, which effect may cause ‘risk reduction’, but there is also a possible facilitative effect between predators acting in different strata and/or having different predatory tactics^55^ leading to a potential ‘risk enhancement’ effect. According to a semi-field study, the MPE of spiders on grasshoppers and on plants was the average of the individual predator effects, while under warmer conditions spider microhabitats overlapped more, and predominant intraguild predation largely reduced the system to a single predator system^21^. While it cannot be directly deduced from the present substitutive experimental design^56^, the differential thermal performance of the two predators may also alter intraguild consumptive and non-consumptive effects. We suggest that studying a diversity of predators, where the species represent a range of thermal responses offers a potentially fruitful future research direction.

The interaction of temperature and predator presence caused activity changes in the cucumber beetles both in their choice of stratum in the mesocosm and in their feeding activity. Concurrently dealing with higher temperature and predator presence meant opposite pressures on beetle behaviour for both aspects of activity. Presence of the ground active lycosid spider caused about two-thirds of beetles to stay higher on the plant at ambient temperatures, whereas without spider or when a canopy active pisaurid was present most beetles stayed at the lower stratum. However, this antipredator response against the lycosid was overridden at higher temperatures, which compelled virtually all beetles to choose the lower stratum. This is a likely case of a trade-off between antipredator behaviour and environmental constraints^57, 58^. For cucumber beetles avoiding heat stress appeared to be a more immediate need than lowering predator encounter probability, presumably because failing to avoid heat stress would have meant an exceedingly high risk of mortality. Concurrently acting multiple stressors often involve an internal conflict between alternative options^59^. Trade-offs between foraging activity and predator avoidance have been more in the focus of recent studies^60, 61^, but review of field studies have proved the context sensitivity of NCEs^62^. Studying the effect of weather variability, it became apparent that foraging-predation risk trade-offs should be combined with survival trajectories contingent on environmental factors to successfully explain prey behaviour and its ecosystem-wide effects^63^.

Higher metabolism and consequent higher energy demand at higher temperature is another need that has to be balanced against predation risk. In the spider – cucumber beetle system the behavioural observations demonstrated that cucumber beetles reacted to predation and heat stress by altering their feeding behaviour, with feeding activity being higher at the high temperature treatment. However, there was an interaction between spider and temperature effect, more pronounced for the pisaurid spider, meaning, that at lower temperature feeding activity decreased in the presence of spiders, whereas at higher temperature the effect was opposite. Higher overall feeding activity despite the presence of spiders can be explained similarly to the stratum choice, by prioritizing physiological needs over predator avoidance. Similar results were found in other herbivorous insects, for instance, stress caused by starvation altered antipredator response in the aphid *Acyrthosiphon pisum* ^64^. Hunger also interacted with predator avoidance in intraguild predation situation^65^. However, the present (marginally significant) interaction suggests, that at the high temperature the proportion of beetles recorded as feeding showed a different tendency when predator was present. The proportion of beetles was actually greater in the high temperature predator treatments than in the high temperature control. We think that more frequent feeding does not necessarily signify a more risk prone behaviour; on the contrary, it might indicate that vigilance lengthened the feeding process resulting in a higher proportion of beetles observed feeding during the hourly observations. Optimal foraging theory predicts that with increased encounter rate with predators patch use behaviour increases while its efficiency decreases^66^, which supports that more frequent feeding may translate into actually less food intake. An empirical example for this phenomenon is a mesocosm study where, under continuous recording of behaviour, in the presence of spider predator leafhoppers’ foraging movements became more frequent but also more brief^67^.

As heat results in increased metabolism and consequent demand for higher food intake^47^, we observed that plant damage was always higher in the elevated temperature treatment. Adding spiders to the system lowered plant damage only at high temperature, but had no significant effect at the lower temperature. This shows that predators, while they might be relatively inefficient at normal temperatures, may interrupt more intensive feeding in warmer situations. It is interesting to decompose plant damage changes and try to attach relative importance to predator effect through mortality and eliciting activity changes in the beetles. As we have seen, mortality by the lycosid predator was pronounced at both temperatures, the pisaurid predator caused only slight mortality at the high temperature, and spider presence at high temperature did not lead to decreased feeding activity in beetles. Thus, the reduction in plant damage at the higher temperature can be only partially explained by predation. Especially in the case of the pisaurid spider NCEs must have played an important role. At lower temperatures the failure of direct predation to translate into lower plant damage could be seen as an indication that NCEs were maybe less pronounced at the lower temperature treatment. A supplementary experiment was also performed (see Supplementary information for its description and results), in which predator treatment was staged with spiders with their chelicerae glued together. This experimental design provided additional evidence for the existence of NCE, since plant damage inflicted by the beetles was lower in the presence of spiders incapable of predation. Overall, the results suggest that NCEs are constituents of a full-scale cascading predator effect that influences further levels of the food web. The important role of non-consumptive effects shaping ecosystems has already gained recognition^68^. Meta-analytic results suggest that increasing predation risk has a large negative effect on the amount of food consumed by prey, but effect sizes in the studies were heterogeneous^69^. Overall, in the present study increased plant consumption at high temperature was reduced by c. 50% by either spider predator, which are similar values reported by other studies^27, 28^.

Overall, our results suggest that warming radically changes herbivory and the effect of predators on herbivores in our system modelling organically grown cucumbers. Predators could not effectively reduce herbivory by cucumber beetles at normal temperatures, but in the warming treatment they reduced plant damage by c. 50%. Predator caused mortality could not alone explain this reduction. The effect of spiders with glued chelicerae supported that non-consumptive effects could serve as explanation for the missing component of predator effect. The two spider predators had different thermal tolerances, which resulted in different rates of predation at the investigated temperatures, but NCEs compensated for this, and eventually both predators had similar effects on plant damage at both temperatures. Elevated temperatures pose an important problem both due to predicted climate change effects and because of heat accumulating covers applied widely in organic cultures. It can be challenging to grow organic cucurbits due to the difficulty of fighting pests while simultaneously promoting natural enemies. If warming is limited (for which management options exist) the complementarity of predators’ heat tolerances may offer biological control over a wider temperature range, and the mode of predator effect (consumptive vs. non-consumptive) may also complement each other. The present results underline the effectiveness of generalist arthropod predators in such systems. The interaction between the thermal environment and the modes of predator effect found here, shows the necessity to study this potential interaction in other studies that asses the effect of global warming on ecosystem functioning.

## Electronic supplementary material

Supplementary information

Supplementary dataset 1

Supplementary dataset 2

Supplementary dataset 3
