# Supplementary material for: Natural enemies partially compensate for warming induced excess herbivory in an organic growth system
Source: Sci Rep. 2017 Aug 4;7:7266. doi: 10.1038/s41598-017-07509-w (PMC5544682; doi:10.1038/s41598-017-07509-w)

Supplementary Information

Natural enemies partially compensate for warming induced excess herbivory in an organic growth system

Orsolya Beleznai, Jamin Dreyer, Zoltán Tóth and Ferenc Samu

Supplementary experiment to test for the presence of NCE

We conducted a supplementary experiment to test the presence of NCE with spiders that had their chelicera glued together so that they could not predate on the beetles. The experimental setup was exactly the same as in the tritrophic system. We ran these trials in three replications for the predator treatment × temperature level combinations. This experiment was performed on 2 November 2015.

We calculated the percentage of total plant damage as the average of the assessed percentage of damaged area on the two leaves and the stem. This calculation method is identical to the one used in the tritrophic experiment presented in the manuscript. We fitted a generalized least squares model to examine the effect of temperature (22 °C/38 °C), spider treatment (Control [no predator]/Lycosidae/Pisauridae) and the interaction of these two variables on the inflicted level of plant damage, with varIdent function ('nlme' package[1] in R) to control for different variances in the spider treatment levels. Model assumptions were checked and confirmed by plot diagnosis.

We found that both the temperature (χ^2 =10.29, d.f. = 1, P = 0.001) and the spider treatment (χ^2 =7.10, d.f. = 2, P = 0.029) significantly affected total plant damage: beetles inflicted more damage at high temperature than at low temperature, but tended to be less when spiders were present compared to the control irrespective of the temperature regime (S Figure 2). The interaction between these variables was not significant (P=0.991). This result provides additional evidence that the presence of at least one of the tested spider species was likely to induce non-consumptive effect on the beetles' feeding activity, leading to reduced herbivory in the studied organic growth system. This finding also corroborates with previous studies of NCE on the same prey species (e.g. Snyder & Wise[2]).

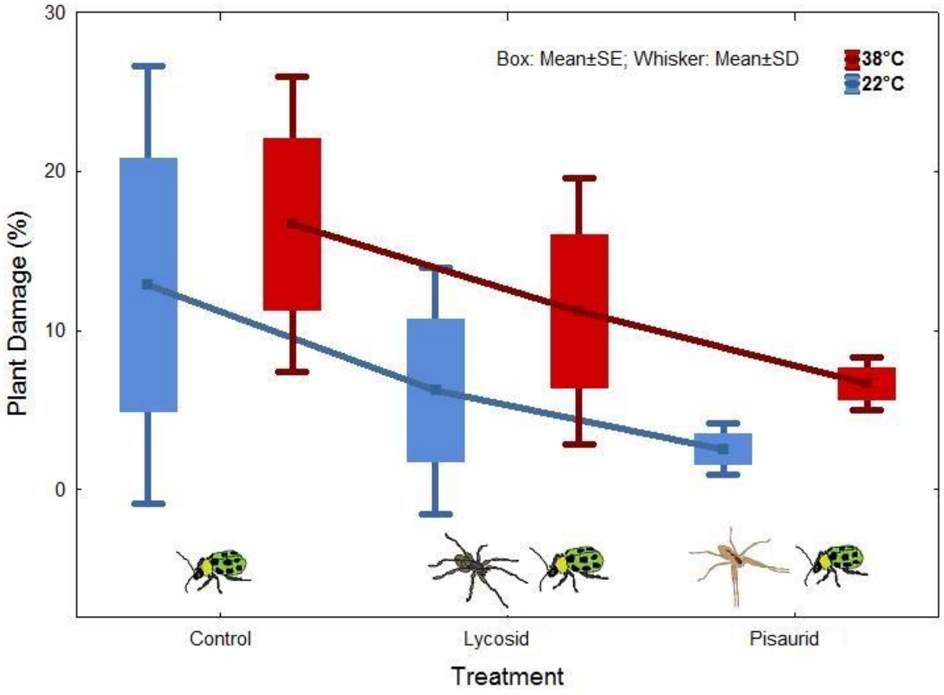

S Figure 2: Cucumber beetle responses. Mean cucumber beetle responses in Plant Damage to the three treatments (control, lycosid, pisaurid) under the two different temperatures (22°C, 38°C). Spiders'chelicerae were glued together. Plant damage is the mean percentage of damage on the two leaves and the stem of cucumber plants by the end of the experiment.

References

1 Pinheiro, J., Bates, D., DebRoy, S., Sarkar, D. & R Core Team. Linear and nonlinear mixed effects models. R package version 3.1-131. (2017).

2 Snyder, W. E. & Wise, D. H. Antipredator behavior of spotted cucumber beetles (Coleoptera : Chrysomelidae) in response to predators that pose varying risks. *Env. Entomol.* **29**, 35-42, doi:10.1603/0046-225x-29.1.35 (2000).

Supplementary Fig. 1: Introduction method of the spider into the mesocosms at ground level. Cylinders were tilted briefly; first beetles were placed into the mesocosms then the spider 15 minutes, as seen on the figure.

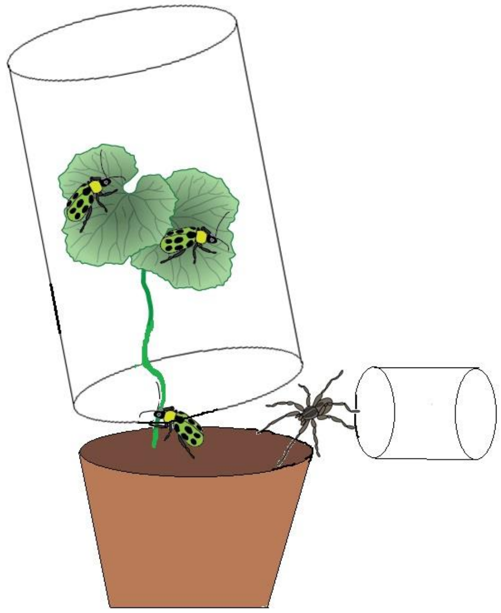

Supplement: Supplementary file 1 — Supplementary information [file 41598_2017_7509_MOESM1_ESM.pdf]
